# Macular Development in Aggressive Posterior Retinopathy of Prematurity

**DOI:** 10.1155/2015/808639

**Published:** 2015-06-18

**Authors:** Hemang K. Pandya, Lisa J. Faia, Joshua Robinson, Kimberly A. Drenser

**Affiliations:** ^1^Kresge Eye Institute, Wayne State University, Detroit, MI, USA; ^2^Associated Retinal Consultants, P.C., Royal Oak, MI, USA; ^3^Associated Retinal Consultants, William Beaumont Hospital, 3535 W 13 Mile Road, No. 344, Royal Oak, MI 48073, USA

## Abstract

*Purpose*. To report anatomic outcomes after early and confluent laser photocoagulation of the entire avascular retina, including areas in close proximity to the fovea, in patients with APROP. We aspire to demonstrate fundoscopic evidence of transverse growth and macular development following laser treatment in APROP. *Methods*. Retrospective review of 6 eyes with APROP that underwent confluent laser photocoagulation of the entire avascular retina. Photographic fundoscopic imaging was performed using the RetCam to compare outcomes after treatment. *Results*. Mean birth weight and gestational age were 704.8 g and 24.33 weeks, respectively. There were 2 females and 1 male. The average time to laser was 9.3 weeks after birth, with the mean postmenstrual age of 34 weeks. Two eyes had zone 1 and 4 eyes had posterior zone 2 disease. Three eyes developed 4A detachments, which were successfully treated. All 6 eyes experienced transverse growth, with expansion of the posterior pole and anterior displacement of the laser treatment. *Conclusion*. Confluent photocoagulation of the entire avascular retina, regardless of foveal proximity, should be the mainstay for treating APROP. Examination should be conducted within 5–10 days to examine areas previously hidden by neovascularization to ensure prudent therapy. Macular development involves both transverse and anterior-posterior growth.

## 1. Introduction

Aggressive posterior retinopathy of prematurity (APROP) is a severe and uncommon form of retinopathy of prematurity (ROP), seen in significantly premature infants of low birth weight (<750 g) and young gestational age (<26 weeks). These eyes are characterized by rapid, progressive vascular changes, flat neovascularization, intraretinal shunting, hemorrhages, and ultimately retinal detachment, despite not progressing through stages 1–3 [1–4]. APROP eyes have a poorer prognosis compared to that of classic ROP eyes, with retinal detachment rates as high as 45% [4–6].

The incidence of APROP has increased, attributable to improved neonatal care and the resulting increase in survival of premature infants [7–9]. Both the Cryotherapy for Retinopathy of Prematurity (CRYO-ROP) [10, 11] and Early Treatment for Retinopathy of Prematurity (ET-ROP) [12, 13] studies demonstrated improved structural and functional outcomes with peripheral ablation of the avascular retina, including the areas in close proximity to any flat neovascularization. When compared to full term neonates, these APROP patients are at a different stage of retinal and vascular development. APROP infants, in particular, may have an avascular “temporal notch” of retinal tissue within zone 1 [14].

A previous study evaluating APROP, using Photographic Screening for Retinopathy of Prematurity (PHOTO-ROP) patients, recognized that unique treatment guidelines did not exist [15]. They suggested that photocoagulation be applied to the avascular peripheral retina including the areas in close proximity to any flat neovascularization. Subsequent examination and photocoagulation should be performed within 10 days, as the neovascular areas will have involuted, thus exposing the previously hidden avascular retina. Laser photocoagulation of the avascular retina has since become the mainstay therapy for APROP. However, due to its proximity to the fovea, there exists a hesitance to apply ablation to the “temporal notch,” particularly for fear of laser spread posteriorly (or “creep”).

Our study aims to report anatomic outcomes after early and confluent laser photocoagulation of the entire avascular retina, including areas in proximity to the fovea, in patients with APROP. In addition, we demonstrate, through fundoscopic evidence, that transverse growth and macular development are seen after the treatment of eyes with APROP.

## 2. Methods

A retrospective case review of infants with APROP seen between January 2004 and January 2008 evaluated and treated at the Associated Retinal Consultants P.C. with confluent laser photocoagulation of the entire avascular retina was performed. These infants were evaluated for clinical course, treatment interventions, and anatomical outcomes. Babies were excluded if they were transferred from an outside hospital and had received previous treatment elsewhere. APROP was characterized as stage 3, zone 1, or posterior zone 2 with Plus or pre-Plus disease.

Evaluations included a dilated fundus examination under anesthesia to assess the extent of APROP. Serial photographic fundoscopic imaging was performed using the RetCam (MLI Inc., Pleasanton, CA) to compare retinal anatomy before and after treatment. All infants underwent confluent peripheral laser ablation of treatment-requiring disease based on ICROP classification [1] and ETROP criteria [12]. Additional therapies including vitrectomy were also performed if necessary. The primary outcome measures were the regression of neovascularization and attachment of the retina.

Institutional review board (IRB) approval was obtained. Written informed consent in accordance with the Declaration of Helsinki was obtained from the parents/guardians of all patients. All procedures were performed by a single vitreoretinal surgeon (KAD) at William Beaumont Hospital, Royal Oak, MI.

## 3. Results

A total of 6 eyes of 3 patients were reviewed with patient demographics listed in Table 1. The average gestational age was 24.3 weeks with a range of 23.6–25.6 weeks. The average birth weight was 705 grams with a range of 525–829 grams. The average time to laser treatment was 9.3 weeks after birth, with the mean postmenstrual age of 34 weeks (range 32–36 weeks). Two eyes had zone 1 disease and 4 eyes had posterior zone 2 disease. Associated comorbidities are noted in Table 1.

All 6 eyes received confluent and complete laser photocoagulation of the entire avascular retina, including areas in close proximity to the fovea. Three eyes demonstrated appropriate regression of neovascular pathology and Plus disease with a single treatment of peripheral laser ablation. There were no cases of anterior segment ischemia, cataract formation, vitreous hemorrhage, or rhegmatogenous retinal detachment. However, 3 eyes developed 4A detachments, which were successfully treated with a lens-sparing vitrectomy, and, at last exam, had grossly normal appearing posterior poles. All tractional retinal detachments occurred near the due date, similar to that seen in classic ROP [14].

The mean follow-up was 7 months (range 3–12 months) and all 6 eyes experienced transverse growth, with expansion of the posterior pole and anterior displacement of the previous laser treatment. At last follow-up, all patients were able to fixate and follow.

## 4. Discussion

Aggressive posterior retinopathy of prematurity (APROP) is a vitreoretinal abnormality that adversely affects premature babies with extremely low birth weights. APROP rates have increased over the last 30 years and continue to be a significant cause of childhood blindness [16]. Our recognition of this debilitating ocular disease and the hesitation of some of our colleagues to perform confluent laser in these patients prompted our review of these infants and their macular growth.

**Figure 1:**
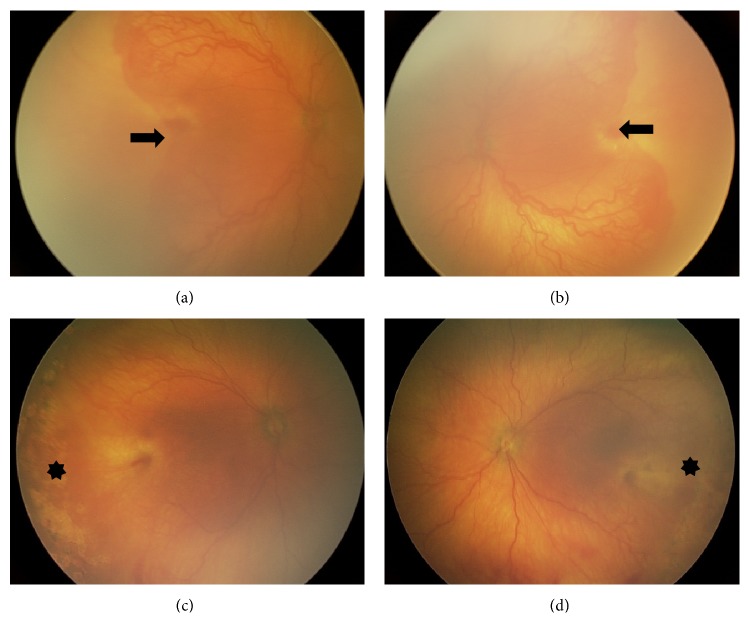


**Figure 2:**
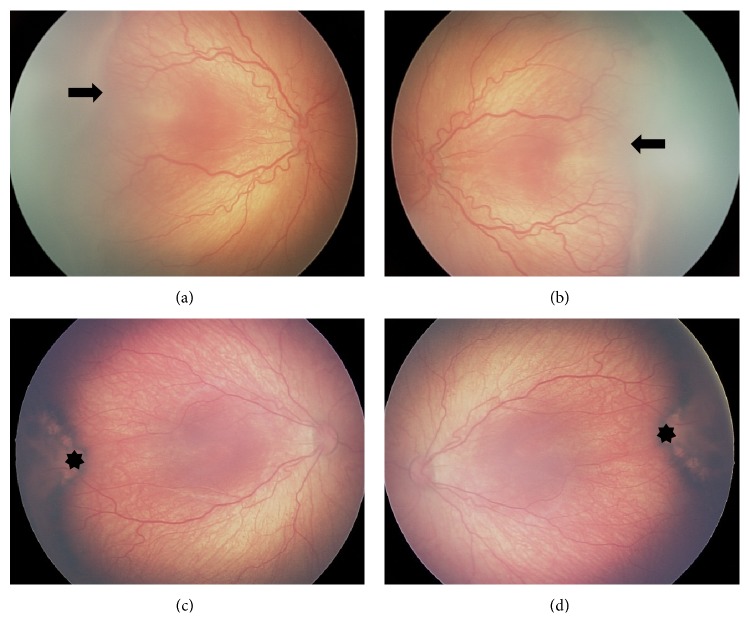


**Figure 3:**
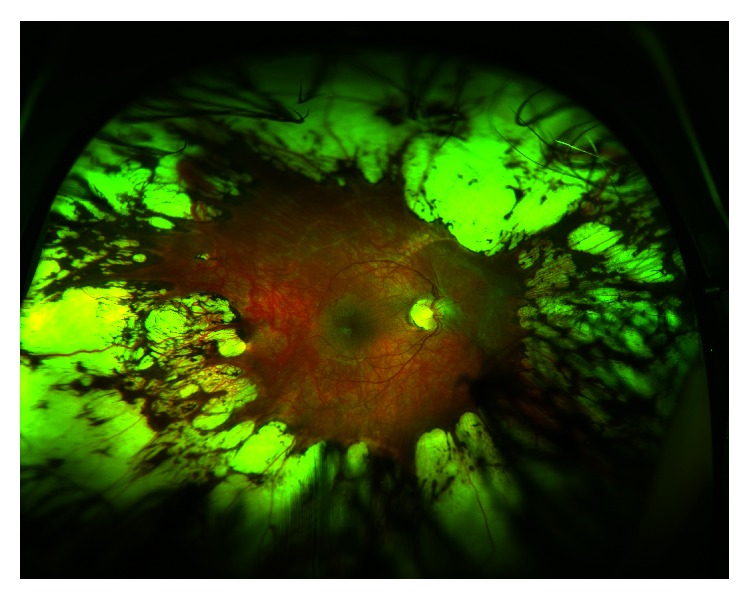


In all six of our treated eyes, there was no evidence of posterior creep. In fact, our serial photography demonstrated evidence of anterior displacement of the applied confluent laser. Four representative eyes from two patients—one with zone 1 disease and one with posterior zone 2 disease—are shown in Figures 1 and 2. In addition, Figure 3 contains wide-field fundus photography of a 7-year-old patient who initially presented with zone 1 disease (Figure 3). Interestingly, the patient's best-corrected visual acuity was 20/40, macula was flat, and all applied confluent laser photocoagulation scars were displaced anteriorly.

A wedge-shaped area of temporal avascular retina, in close proximity to the fovea, is classically seen in APROP. A previous study asserted that this “temporal notch” arises from the geometry of the vascular ellipses as they move apart and lose any overlap [17]. In addition, we postulate that a developmental lag contributes to the forming of this temporal notch, as transverse growth in our patients is demonstrated by anterior displacement of the laser treatment.

The macula is characterized by a high cell-to-signal ratio. The metabolic demand of this highly concentrated tissue is greater than in the remainder of the zone 1 retina and may be involved in the forming of the temporal notch that characterizes APROP. A previous neonatal study, which evaluated the macular development in premature infants [18] using a direct ophthalmoscope concluded that the fovea matures 3 months after the target due date. Unfortunately, the mechanisms and nature of macular development were not characterized.

We propose that macular development and ocular maturation involve 2 distinct processes: (1) retinal stretch (transverse growth) and (2) anterior-posterior (AP) retinal layer growth. The fundoscopic evidence presented in Figures 1 and 2 shows that the initial laser scars appeared more anteriorly with maturation. We believe that transverse and AP retinal layer growth and remodeling contribute to maintaining a healthy macula in laser-treated APROP patients, although additional prospective investigation is warranted.

Our study is limited by its size and retrospective nature. Still, these patients clearly demonstrated transverse and AP growth as evident by the serial photography. In order to better explore this observation, future studies will not only focus on obtaining larger numbers but also incorporate observations of macular development using premature mammal models and optical coherence tomography (OCT) evidence of retinal layer changes. Recently, a study by Vajzovic et al. utilized SD-OCT to evaluate and compare retinal morphology with histological slides from comparable ages [19]. Further studies evaluating macular development using SD-OCT are strongly encouraged.

In recent years, antivascular endothelial growth factor (VEGF) agents have shown to be a useful adjuvant in the treatment of retinopathy of prematurity [20]. However, the long-term systemic effects in these prematurity infants remain unknown. Although it did not reach statistical significance, there was increased morbidity in the infants treated with bevacizumab compared to laser in the BEAT-ROP trial [21]. Additionally, VEGF is required for neurodevelopment and VEGF blockade in zone 1 development may adversely affect retinal development in the premature neonate, as seen in animal models of ROP [22]. Confluent photocoagulation of the entire avascular retina, regardless of foveal proximity, should be the mainstay for treating APROP. A thorough examination and possible subsequent photocoagulation should be conducted within 10 days to areas previously hidden by neovascularization to ensure prudent therapy.

## Figures and Tables

**Table 1:** Patient demographics and clinical information.

Patient	Gender	Race	Gestational age (weeks)	Birth weight (grams)	Comorbidities	Initial ROP staging	Initial Plus disease	Initial laser (weeks)
1	Male	African- American	23 6/7	760	Hydrocephalus s/p ventriculoparietal shunt, bowel perforation	OD: stage 3, zone 1 OS: stage 3, zone 1	OD: 2-3+ OS: 2-3+	PRP OU @ 33 5/7

2	Female	Caucasian	23 4/7	525	Pulmonary hypertension, conductive hearing loss	OD: stage 2, zone 2 OS: stage 2, zone 2	OD: 2-3+ OS: 2-3+	PRP OU @ 36

3	Female	Caucasian	25 4/7	829	Pulmonary hypertension	OD: stage 3, zone 1 OS: stage 3, zone 1	OD: 2-3+ OS: 2-3+	PRP OU @ 33 5/7
